# Supplementary material for: Asynchronous magnetic resonance elastography: Shear wave speed reconstruction using noise correlation of incoherent waves
Source: Magn Reson Med. 2022 Oct 27;89(3):990–1001. doi: 10.1002/mrm.29502 (PMC9792433; doi:10.1002/mrm.29502)
Supplement: Supplementary file 1 — DATA S1 MATLAB code used to generate the numerical simulations of Figure 1 [file MRM-89-990-s002.zip › k-Wave/helpfiles/calculateMassSourceCW.html]

calculateMassSourceCW :: Functions (k-Wave)


# calculateMassSourceCW

Compute k-Wave input plane from measured CW data.

## Syntax

```
source_estimate = calculateMassSourceCW(measured_data, dx, freq, c0, source_offset)
source_estimate = calculateMassSourceCW(measured_data, dx, freq, c0, source_offset, grid_expansion)
source_estimate = calculateMassSourceCW(measured_data, dx, freq, c0, source_offset, [], ...)
source_estimate = calculateMassSourceCW(measured_data, dx, freq, c0, source_offset, grid_expansion, ...)
[source_estimate, output] = calculateMassSourceCW(measured_data, dx, freq, c0, source_offset)
...
```

## Description

`calculateMassSourceCW` takes a measured 2D plane of complex pressure values (e.g., measured using a hydrophone in a scanning tank under steady state conditions) and calculates an equivalent CW additive pressure source positioned a given distance away that recreates the measured data when used as an input to k-Wave (i.e., by assigning the equivalent source as an input to `acousticFieldPropagator`, or by generating a time-varying input signal using `createCWSignals` and assigning this to `source.p` with `source.p_mode = 'additive'`). The measured data is assumed to be for a single-frequency continuous wave source, specified by `freq`, and is given as a complex 2D matrix.

The equivalent source is calculated using an iterative optimisation based on gradient descent, where functional gradients are calculated using the adjoint. Both the forward and adjoint operators are computed using `acousticFieldPropagator`. The calculation assumes the propagation is linear and the medium is lossless. The algorithm and approach are described in detail in [1].

If the source is larger than the measured input plane, for example, if measuring a focused bowl transducer, a suitable value should be specified for the value of `grid_expansion`. Note, the value of `source_offset` does not need to match the position of the real source in the experiment.

An alternative approach to project measured data using k-Wave is to directly use the measured data as a pressure source with a Dirichlet boundary condition (i.e., by generating a time-varying input signal from the measured data using `createCWSignals` and assigning this to `source.p` and setting `source.p_mode = 'dirichlet'`). However, this approach leads to errors in the imposed spatial gradient, which manifests as errors in the projected field. Thus, for accurate holographic projections using k-Wave, it is recommended to use `calculateMassSourceCW` to first calculate the input data (see [1] for a comparison).

[1] Treeby, B., Lucka, F., Martin, E., & Cox, B. T. (2018). Equivalent-Source Acoustic Holography for Projecting Measured Ultrasound Fields Through Complex Media. IEEE Transactions on Ultrasonics, Ferroelectrics, and Frequency Control, 65(10), 1857-1864.

## Inputs

|  |  |
| --- | --- |
| `measured_data` | 2D matrix of complex pressure values [Pa]. |
| `dx` | Spatial step between grid points in the input plane [m]. |
| `freq` | Source frequency [Hz]. |
| `c0` | Speed of sound in the medium [m/s]. |
| `source_offset` | Offset between the measured input plane and the source plane [grid points]. For example, if `source_offset = 1`, the input plane and source plane are on adjacent grid points. |
| `grid_expansion` | Number of grid points used to expand the size of the estimated source plane in each lateral dimension relative to the measured input plane (set to 0 if not defined). |

## Optional Inputs

Optional 'string', value pairs that may be used to modify the default computational settings.

| Input | Valid Settings | Default | Description |
| --- | --- | --- | --- |
| `'NumSteps'` | *(integer)* | `20` | Number of gradient descent steps. |
| `'StepSize'` | *(numeric scalar)* | `0.5` | Starting size of gradient descent step. |
| `'StepSizeIncrement'` | *(numeric scalar)* | `1.1` | Multiplicative factor used to increase the step size when the error is reduced. |
| `'StepSizeDecrement'` | *(numeric scalar)* | `0.5` | Multiplicative factor used to decrease the step size when the error is increased. |
| `'Plot'` | *(Boolean scalar)* | `true` | Boolean controlling whether the update steps are displayed. |
| `'ReturnIterations'` | *(Boolean scalar)* | `false` | Boolean controlling whether the source estimate at each gradient descent step is returned. If set to `true`, the `source_estimate` output is given as a 3D matrix, each x-y plane corresponds to the source estimate at each step. |
| `'UseCpp'` | *(Boolean scalar)* | `false` | Boolean controlling whether the simulations are run using the C++ implementation of `acousticFieldPropagator`. |

## Outputs

|  |  |
| --- | --- |
| `source_estimate` | If `'ReturnIterations'` is `false` (the default), `source_estimate` is given as a 2D matrix of complex pressure values [Pa]. If `'ReturnIterations'` is `true`, `source_estimate` is given as a 3D matrix containing the source estimate after each iteration, indexed as (x, y, iteration). |
| `output` | Structure containing details of the optimisation with the following fields: |
|  | `.linf_error` |
|  | `.l2_error` |
|  | `.step_size` |
|  | `.number_steps` |
|  | `.number_function_calls` |
|  | `.modelled_data` |

## See Also

`acousticFieldPropagator`, `calculateMassSource`
